# The chicken or the egg? *Mycoplasma pneumoniae* complicated by left ventricle thrombus and anterior myocardial infarction: a case report

**DOI:** 10.1093/ehjcr/ytae434

**Published:** 2024-08-22

**Authors:** Nina Balac, Kyle F Nelson, Tara Naib, Ahmed El-Eshmawi, Martin E Goldman

**Affiliations:** Icahn School of Medicine at Mount Sinai, Department of Internal Medicine, One Gustave Levy Place, New York, NY 10029, USA; Icahn School of Medicine at Mount Sinai, The Zena and Michael A. Wiener Cardiovascular Institute, New York, NY 10029, USA; Icahn School of Medicine at Mount Sinai, The Zena and Michael A. Wiener Cardiovascular Institute, New York, NY 10029, USA; Icahn School of Medicine at Mount Sinai, Department of Cardiovascular Surgery, New York, NY 10029, USA; Icahn School of Medicine at Mount Sinai, The Zena and Michael A. Wiener Cardiovascular Institute, New York, NY 10029, USA

**Keywords:** Left ventricle, Myocardial infarction, Thrombus, Systemic embolism, Thrombectomy, Case report

## Abstract

**Background:**

*Mycoplasma pneumoniae* can be associated with extrapulmonary manifestations, including vasculitis, myocarditis, and thrombosis. In rare cases, it has also been implicated in intracardiac thrombus formation.

**Case Summary:**

A previously healthy 25-year-old male presented with worsening abdominal pain, an episode of acute chest pain, new lightheadedness, and gait instability in the setting of *M. pneumoniae*. Initial blood tests were notable for mild coagulopathy, thrombocytosis, transaminitis, and elevated high-sensitivity troponin. Further, workup revealed systematic emboli to the cerebellum, kidneys, spleen, anterior myocardial infarction, and a left ventricular multilobular mural mass. Due to the unknown composition of the mass with concern for further embolic events, the patient underwent successful surgical excision with the mass ultimately defined as a thrombus. Hypercoagulable workup was notably inconclusive and intraoperative myocardial biopsies revealed organizing infarction without inflammation or healed myocarditis. Post-operative course was complicated by left ventricular dysfunction and acute kidney injury, both with eventual improvement. Patient has remained on guideline-directed medical therapy and prophylactic anticoagulation.

**Discussion:**

We presume that the formation of the ventricular thrombus in this case was a result of transient thrombophilia in the setting of *M. pneumonia* resulting in coronary obstruction and subsequent myocardial injury. This case underscores the challenge of determining the pathophysiological sequence of events in patients with mycoplasma who develop systemic embolism and the management of a large residual thrombus, particularly in regard to surgical consideration.

**Learning points**

- Recognize intracardiac thrombus as a rare, but reported complication of *Mycoplasma pneumoniae*, associated with severe inflammation, particularly in younger patients.
- Understand the potential mechanisms and options for management of thrombotic complications in this situation.

## Introduction

*Mycoplasma pneumoniae* has been associated with extrapulmonary manifestations including vasculitis, myocarditis, and thrombosis. The formation of ventricular mural thrombus is rare with case reports involving mainly paediatric patients.^1–7^ Multiple pathophysiological mechanisms have been proposed to explain thrombosis in patients with *M. pneumoniae*. The inflammatory response to this organism may produce autoantibodies (typically anticardiolipin or lupus anticoagulant antibodies) leading to a transient hypercoagulable state. Mycoplasma can also cause endothelial injury resulting in local vasculitis and activation of the complement pathway, cumulating in thrombus formation.^4,8,9^

## Summary figure

**Figure:**
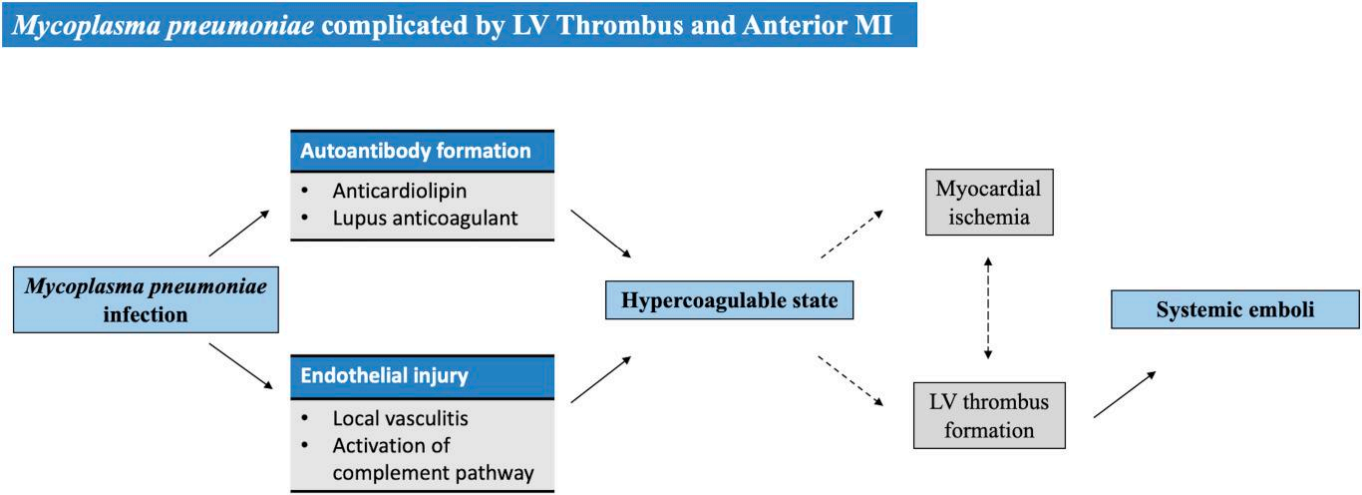


We report a case of *Mycoplasma pneumoniae* complicated by extrapulmonary manifestations involving multiple organ systems including intracardiac thrombus formation.

## Case presentation

A previously healthy 25-year-old male presented to the emergency department with abdominal pain and emesis. Two weeks earlier while abroad, he developed fever, nausea, and shortness of breath. Polymerase chain reaction testing identified *M. pneumoniae*, prompting treatment with azithromycin, doxycycline, and a brief course of corticosteroids. One week later, while on a flight home, he experienced acute, severe left-sided chest pain lasting several hours. Chest pain resolved, but fever and gastrointestinal symptoms continued, and he developed lightheadedness and gait instability. He was treated empirically for viral gastroenteritis, but over the next 2 days his abdominal pain intensified and he developed intractable vomiting.

Upon arrival, he appeared uncomfortable. He was afebrile; heart rate was 104 beats/min, blood pressure 143/96 mmHg, respirations 30 breaths/min, and transcutaneous arterial oxygen saturation was 98% while breathing room air. The lungs were clear to auscultation. The abdomen was guarded and tender in the epigastrium and left upper quadrant. The patient reported no notable medical history or family history of thrombophilia. He worked long hours in finance and prior to his present illness he took no medications regularly. He had never smoked, seldom drank alcohol, and denied any recreational drug use.

**Figure 1:**
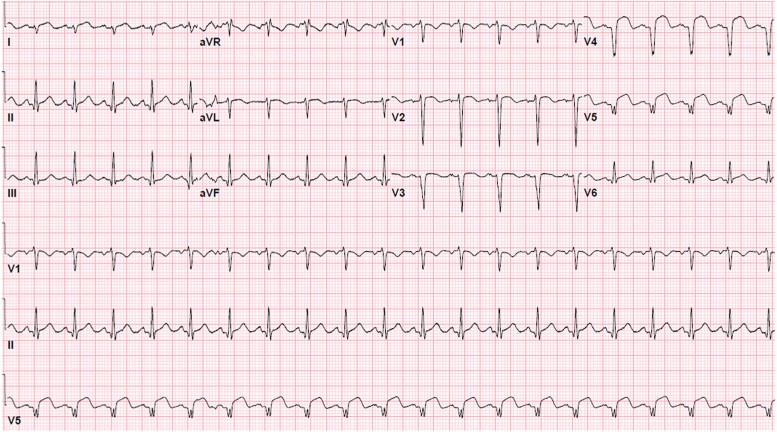
Initial electrocardiogram. ST-segment elevations in the lateral precordial leads.

Initial laboratory findings were notable for leukocytosis [22.2 × 10^3^/µL, reference range (RR) 4.5 – 11 × 10^3^/µL], thrombocytosis (522 × 10^3^/µL, RR 150 – 450 × 10^3^/µL), and mild transaminitis (alanine transaminase 189 U/L, RR 1 – 45 U/L and aspartate transferase 71 U/L, RR 1 – 35 U/L). The prothrombin time was mildly prolonged (14.7 s, RR 12.3 – 14.9 s) as was the partial thromboplastin time (40.5 s, RR 25.4 – 34.9 s). There was respiratory alkalosis (venous blood pH 7.50, RR 7.33 – 7.43) and elevated blood lactate (4.10 mmol/L, RR 0.50–1.99 mmol/L). The high-sensitivity troponin was elevated to > 3600 ng/L (RR < 35 ng/L) and subsequent electrocardiogram showed ST-segment elevation in leads V4 – V6 (*Figure 1*).

**Figure 2:**
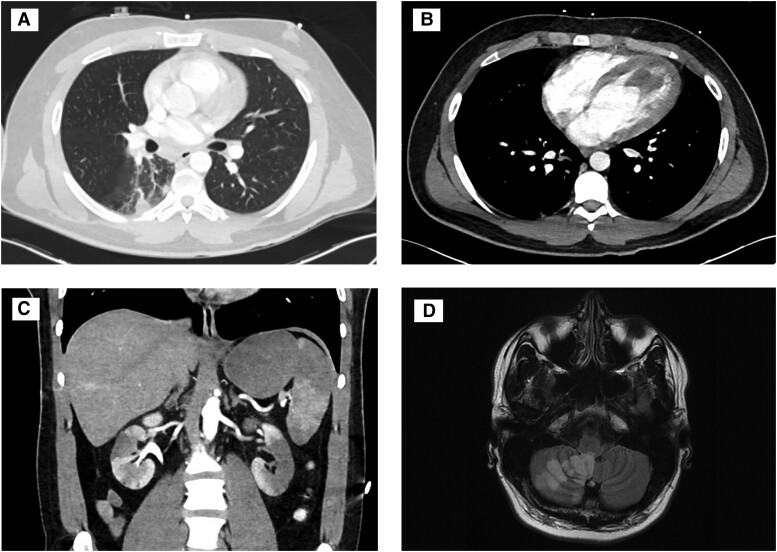
Diagnostic imaging findings. Computed tomography angiography of the chest revealing (*A*) patchy pulmonary opacities, (*B*) apical left ventricular filling defect with hypoattenuation of the apical wall, (*C*) computerized tomographic abdomen and pelvis with areas of hypoattenuation in the kidneys and spleen consistent with embolic infarction, and (*D*) brain magnetic resonance imaging revealing acute infarct of the right inferior cerebellar hemisphere.

Further workup with contrast-enhanced computerized tomographic (CT) imaging revealed patchy pulmonary opacities most prominent at the right lung base, a large (3.6 × 1.8 cm) filling defect in the left ventricular (LV) apex, hypoattenuation of the apical wall adjacent to the filling defect with myocardial thinning, and abnormal enhancement of the spleen and kidneys suggestive of infarction. A CT scan of the head without contrast disclosed acute cerebellar infarction without hemorrhage, best visualized on brain magnetic resonance imaging (MRI) (*Figure 2*). Transthoracic echocardiography found the LV ejection fraction (EF) 35% – 40% with hypokinesis of the anteroseptal, septal, and apical segments and a multilobular mass measuring 11 cm^2^, attached to apex (see Supplementary material online, *Video S1*). Furthermore, catheter-based coronary angiography was done and showed tapering and occlusion of the mid-to-distal portion of the left anterior descending (LAD) coronary artery without visible thrombus; the remainder of the coronary arteries appeared normal (see Supplementary material online, *Video S2*). In addition to aspirin and ticagrelor, heparin was administered by intravenous infusion.

**Figure 3:**
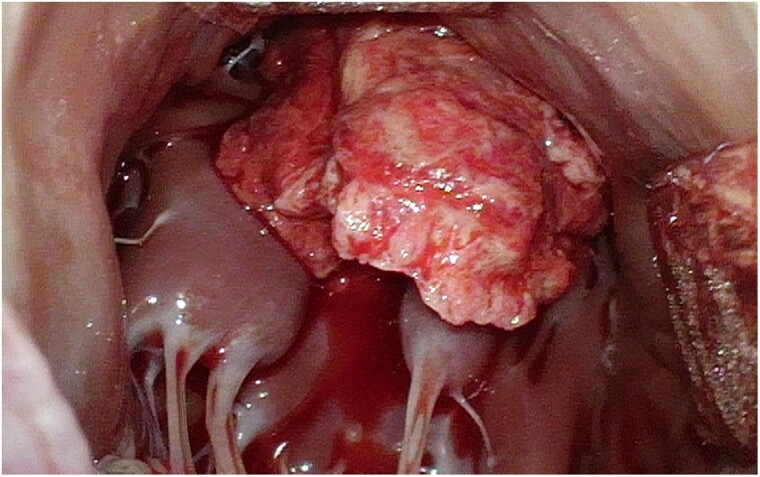
Left ventricular thrombus. Intraoperative photograph.

At this point, the identity of the intracardiac mass remained unclear with differential including both thrombotic and non-thrombotic causes, the latter group including cardiac tumours such as myxoma, fibroelastoma, angiosarcoma, lymphoma, or vegetation, either infective or non-infective (*e.g.* verrucous). Additionally, given that the size and appearance of the mass raised the risk of recurrent embolism, anticoagulation was not deemed sufficiently protective and systemic thrombolytic therapy was contraindicated in the setting of a recent embolic stroke. Therefore, the patient underwent surgical exploration via a transaortic approach under cardiopulmonary bypass (*Figure 3*, Supplementary material online, *Video S3*). The mass proved thrombotic and was directly excised. Bypass of the obstruction in the LAD artery was not pursued after visual inspection of the myocardium suggested completed infarction and no epicardial coronary plaque or other arterial lesion was identified. Myocardial biopsies found organizing infarction without inflammation or healed myocarditis.

The post-operative course was complicated by LV dysfunction (EF 24%), left bundle-branch block, and acute kidney injury. Testing for thrombophilia was inconclusive. Anticoagulation was resumed postoperatively and metoprolol succinate was started. The patient was discharged on apixaban, and upon recovery of renal function, empagliflozin and sacubitril/valsartan were added. Cardiac MRI five months postoperatively showed improvement in EF to 45% with evidence of transmural infarction involving the apex but no residual thrombus. There was focal late intramyocardial gadolinium enhancement in the anterolateral segment, possibly a sequela of myocarditis.

## Discussion

This patient developed LV thrombus, antero-apical myocardial infarction (MI), and systemic emboli in the setting of infection with *M. pneumoniae*, which has been associated with extrapulmonary manifestations. As in previously reported cases, our patient developed anticardiolipin antibodies, supporting the proposed mechanism of thrombus formation in patients with *M. pneumoniae* involving autoantibody formation.^1–4,6^ Although myocardial biopsies did not identify inflammation, non-homogenous, patchy myocarditis could not be excluded.

A particularly puzzling aspect of this case is the sequence of events linking impaired coronary perfusion, MI, and systemic embolism. Given his youth, the angiographic finding of obstructive disease limited to the LAD raised the possibility of spontaneous coronary artery dissection as the cause of acute MI, but his gender and absence of associated fibromuscular dysplasia, aneurysms, or arterial tortuosity pointed away from that mechanism, and intraoperative inspection did not identify coronary artery dissection.^10^ Coronary vasospasm complicated by arterial thrombosis that resolved with anticoagulation cannot be excluded but is speculative. There are multiple causes of LV mural thrombus formation and subsequent systemic emboli including acute MI involving the ventricular apex, embolism originating from the left atrial appendage in patients with atrial fibrillation or valvular heart disease, or secondary to cardiomyopathy or myocarditis. Here we presume that thrombophilia arising in the course of *M. pneumoniae* was involved in both the coronary obstruction that caused myocardial injury and in the formation of ventricular mural thrombus.

Treatment of patients with intracardiac thrombus includes systemic anticoagulation, thrombolysis, or thrombectomy. Typically, the risk of embolism is related to thrombus size, location, and mobility. As described previously, surgical excision was elected in this case. The literature describes treatment of patients with cardiac thrombus associated with *M. pneumoniae* with unfractionated heparin or a low molecular weight heparin, with some transitioning to rivaroxaban. Treatment duration has ranged from days to months to either decreased thrombus size, complete resolution, or improvement in LV systolic function as determinant.^1,3,6–8^ In our patient, selection of a direct oral anticoagulant was based on inability to reliably assess conventional coagulation assays, which were persistently elevated, presumably due to the antibodies associated with *M. pneumoniae,* described above.

In summary, intracardiac thrombus formation is a rare but dangerous complication of *M. pneumoniae* that can be associated with systemic embolism. In patients with mycoplasma infection who develop extrapulmonary findings, cardiac evaluation should be considered. The role for prophylactic anticoagulation must be individualized, but if intracardiac thrombus is identified aggressive management may be necessary to reduce the risk of embolism.

## Supplementary Material

ytae434_Supplementary_Data

## Data Availability

No new data were generated or analysed in support of this research.

## Lead author biography

Dr Nina Balac is completing her chief year of Internal Medicine residency at Mount Sinai Hospital in New York City. She plans to continue her training by pursuing a Cardiology fellowship. Her main area of interest is heart failure.
